# Breach of Medical Etiquette

**Published:** 1908-12-05

**Authors:** 


### Medico-Legal Points.

### BREACH OF MEDICAL ETIQUETTE.

In the case of Neale *v.* The Bartitsu Light Cure Institution, Ltd., which came before Mr. Justice Grantham and a special jury on November 19 and 20, the plaintiff, Dr. Albert Ezra Neale, a doctor of medicine and bachelor of surgery, brought an action against the defendants to employ him as consulting medical superintendent at the defendant's institute at 1 Albemarle Street. As a result of seeing an advertisement the plaintiff was invited to join the defendant's institute as medical superintendent, and being a member of the British Medical Association, he submitted the defendant's proposals to the Ethical Committee, and accordingly certain stipulations were inserted in the agreement with the defendants, made on May 13, 1907. By this agreement plaintiff was engaged for two years at a salary of £350, to be increased later to £400, together with a consultation fee of £1 1s. for every patient introduced by him. The plaintiff was to be instructed in the use of the defendants' electrical machines and appliances, so that he might become qualified to administer medical treatment to the defendants' patients. The plaintiff undertook not to be interested in any other similar business within one hundred miles. The agreement concluded: "The following provisions, imposed by the British Medical Association, are to be adhered to by the parties hereto:—(*a*) The medical superintendent shall have entire control of treatment; (*b*) The medical superintendent shall be solely responsible for regulations and arrangements for admission and dismissal of patients, provided fees are paid; (*c*) No advertisement of any kind to be issued respecting the institute without the knowledge and consent of the medical superintendent." Shortly afterwards the defendants desired to induce the medical profession to send their patients to them, and asked the plaintiff to edit a pamphlet to be sent round to doctors. For that purpose he was handed a red pamphlet, printed in 1906, containing testimonials, and he then learned for the first time that whatever the policy of the institute then was, it had originally been run by a medical charlatan. Plaintiff complained bitterly of having been deceived to Mr. Barton-Wright (the managing director), who said that it had only been sent to a few hunting men, and would not go out again. On plaintiff's return from a holiday in September, 1907, he found that the defendants had had no less than 10,000 copies of the pamphlet printed with additions, and that an agency was distributing them broadcast. It was headed: "A few testimonials. Mr. Barton-Wright states, from practical experiences in an extended private practice, that results obtained justify him in stating that absolute cures can practically be guaranteed in all those cases in the list of ailments enumerated on page 3 which are marked with asterisks, and, further, the treatment possesses the indisputable advantage of being absolutely painless." One of the testimonials ran:—"After suffering from a most obstinate case of chronic sciatica for twenty months and getting absolutely no benefit from medical advice, I was induced to try the Bartitsu Treatment. Before I consulted Mr. Barton-Wright I could not walk one hundred yards without being utterly exhausted, and was never free from pain; however, after a course of treatment I am now able to enjoy a walk of several miles. I cannot speak too highly of the kindness shown to patients at the Bartitsu Institute, and to Mr. Barton-Wright I owe a special debt of gratitude for his success in establishing a cure when everything else had failed." This was preceded by a notice in heavy type as follows:—"This lady came to me in an utter state of exhaustion and despair, and would not believe that she could be cured. She had evidently exhausted every channel in the way of what is recognised as the best medical advice, and had even had recourse to a well-known medical specialist, who professes to cure by hypnotism, all without a vestige of success, and not even of improvement. A well-known neurologist, who also attempts to administer electric treatment, had so far frightened her by giving her shocks that it was months before she could be induced to come near me, in spite of the pain she was suffering, as naturally she thought I should give her shocks also. However, to convince her that she must not class my electrical knowledge and methods with what she had tried before, I undertook to absolutely cure her for a fixed fee, and have cured her." Then followed a list of cases in which cures were guaranteed in from one and a half hours to three weeks. On becoming acquainted with these facts the plaintiff communicated with the Medical Defence Union, and a correspondence ensued with the defendants. The plaintiff tried to be present at a board meeting, but was for some time prevented by Barton-Wright. Eventually he succeeded, and it was then for the first time the charges were made against him which were the subject matter of the counter-claim. In other words an attempt was made to induce plaintiff to resign without compensation. In the meantime the plaintiff was in considerable peril, for one of the red pamphlets had found its way into the hands of the British Medical Association, and they, unknown to the plaintiff, were making inquiries of the defendants as to the name of the medical man referred to therein. The defendants now asserted that they were not responsible for the circulation of these pamphlets, which, they said, was the work of Barton-Wright. The defendants alleged that the plaintiff had connived at the sending out of this pamphlet. Dr. Neale said that had he not found out in time what was being done, he would have run a great risk of being struck off the Medical Register for associating with this man. He denied all the charges made against him by the defendants of negligence and want of skill. Mr. Barton-Wright, the managing director of the company, said that early in 1907 he was considering the appointment of a medical man to the institute. He met the plaintiff, who represented that he had influence with the medical Press. He had tried to advertise the institute in the *British Medical Journal*, but they would not accept his advertisement, and he wanted the plaintiff's influence with them to get advertisements accepted. The agreement between the plaintiff and the Institute was signed on May 13, but the plaintiff was at work on the premises for a week before that. The institute was sending out 10,000 copies of the first red pamphlet between May 6 and 16; the plaintiff knew about that, and to prevent any difficulty between him and the Medical Council his agreement was post-dated so that he should not appear to have been there until after the circulars had been sent out. Some copies came back through the Dead-letter office and they were left in the waiting-room and handed to inquirers. The plaintiff saw witness hand them out and said he was not supposed to see it. In August and September the business was decreasing and the witness thought further advertising was needed. He tried to induce the plaintiff to go to the *British Medical Journal*, but he would not, though that was the reason for his appointment originally. Witness ordered the red pamphlet on his own account entirely and not on behalf of the company; he did this with the object of increasing business, and so increasing the value of his 7,000 ordinary shares in the company. After the issue the plaintiff came to him and said he had found out that the company had issued the pamphlet, and witness told him it had not, and the other directors knew nothing about it until the board meeting of October 8. Mr. Justice Grantham, in summing up, pointed out that by the agreement the business was to be carried on under the rules of the Medical Association and to be entirely under the plaintiff's control; but the pamphlet of September was issued without the plaintiff's knowledge and evidently transgressed the rules. The jury found a verdict for the plaintiff for £600, and judgment was given accordingly. A stay of execution was refused.

